# The hidden interplay between sex and COVID-19 mortality: the role of cardiovascular calcification

**DOI:** 10.1007/s11357-021-00409-y

**Published:** 2021-07-14

**Authors:** Alberto Cereda, Marco Toselli, Anna Palmisano, Davide Vignale, Riccardo Leone, Valeria Nicoletti, Chiara Gnasso, Antonio Mangieri, Arif Khokhar, Gianluca Campo, Alessandra Scoccia, Matteo Bertini, Marco Loffi, Pietro Sergio, Daniele Andreini, Gianluca Pontone, Gianmarco Iannopollo, Tommaso Nannini, Davide Ippolito, Giacomo Bellani, Gianluigi Patelli, Francesca Besana, Luigi Vignali, Nicola Sverzellati, Mario Iannaccone, Paolo Giacomo Vaudano, Giuseppe Massimo Sangiorgi, Piergiorgio Turchio, Alberto Monello, Gabriele Tumminello, Aldo Pietro Maggioni, Claudio Rapezzi, Antonio Colombo, Francesco Giannini, Antonio Esposito

**Affiliations:** 1 GVM Care & Research Maria Cecilia Hospital, Cotignola, Italy; 2 Cardiovascular Department, ASST Santi Paolo e Carlo, Milan, Italy; 3 IRCCS San Raffaele Scientific Institute, Milan, Italy; 4 Vita-Salute San Raffaele University, Milan, Italy; 5 Azienda Ospedaliero-Universitaria di Ferrara, Cona, FE Italy; 6 Ospedale di Cremona, Cremona, Italy; 7 Centro Cardiologico Monzino IRCCS, Milan, Italy; 8 Ospedale Maggiore, Bologna, Italy; 9 San Gerardo Hospital, Monza, Italy; 10 ASST Bolognini Hospital, Bergamo Est, Seriate, Italy; 11 Parma University Hospital, Parma, Italy; 12 San Giovanni Bosco Hospital, ASL Citta di Torino, Turin, Italy; 13 Università degli Studi di Roma “Tor Vergata”, Roma, Italy; 14 Guglielmo da Saliceto Hospital, Piacenza, Italy

**Keywords:** Sex bias, Sars-CoV2, Lung CT; Cardiovascular calcifications, COVID-19

## Abstract

Recent clinical and demographical studies on COVID-19 patients have demonstrated that men experience worse outcomes than women. However, in most cases, the data were not stratified according to gender, limiting the understanding of the real impact of gender on outcomes. This study aimed to evaluate the disaggregated in-hospital outcomes and explore the possible interactions between gender and cardiovascular calcifications. Data was derived from the sCORE-COVID-19 registry, an Italian multicentre registry that enrolled COVID-19 patients who had undergone a chest computer tomography scan on admission. A total of 1683 hospitalized patients (mean age 67±14 years) were included. Men had a higher prevalence of cardiovascular comorbidities, a higher pneumonia extension, more coronary calcifications (63% vs.50.9%, p<0.001), and a higher coronary calcium score (391±847 vs. 171±479 mm^3^, *p*<0.001). Men experienced a significantly higher mortality rate (24.4% vs. 17%, *p*=0.001), but the death event tended to occur earlier in women (15±7 vs. 8±7 days, *p*= 0.07). Non-survivors had a higher coronary, thoracic aorta, and aortic valve calcium score. Female sex, a known independent predictor of a favorable outcome in SARS-CoV2 infection, was not protective in women with a coronary calcification volume greater than 100 mm^3^. There were significant differences in cardiovascular comorbidities and vascular calcifications between men and women with SARS-CoV2 pneumonia. The differences in outcomes can be at least partially explained by the different cardiovascular profiles. However, women with poor outcomes had the same coronary calcific burden as men. The presumed favorable female sex bias in COVID-19 must therefore be reviewed in the context of comorbidities, especially cardiovascular ones.

## Introduction

The SARS-CoV2 outbreak has had a significant impact on healthcare services and resource allocation. Accumulating evidence has shown that amongst COVID-19-infected patients, elevated mortality is observed in older patients and those with pre-existing comorbidities, including hypertension, diabetes mellitus, cardiovascular disease, chronic lung disease, and cancer [1–3].

The available sex-disaggregated data reveal that men experience a higher hospitalization rate, a more severe disease than women with a consequent higher overall case fatality ratio [1]. Epidemiological data on sex differences are still under investigation and need to be cleared of potential bias. A better knowledge of sex differences in incidence and mortality in the COVID-19 pandemic is the first step to analyze the biological patterns to define gender-specific prevention and treatment strategies.

The high cardiovascular risk profile of males and females has been proposed to explain the observed gender bias in outcomes.

Recent studies have demonstrated a complex interplay between comorbid cardiovascular disease, COVID-19 pathophysiology, and poor clinical outcomes. Coronary artery calcification (CAC) may therefore aid in risk stratification of COVID-19 patients.

Lung CT scans are obtained in COVID-19 patients to assess the extent of lung disease, monitor disease progression, and investigate the presence of disease complications.

The analysis of vascular calcifications (coronary, aortic, and valvular) can provide a rapid and quantifiable stratification of the cardiovascular profile. This imaging biomarker may provide further insight into the disease process from both pathophysiologic and clinical perspectives, performed in the context of a pandemic with limited health resources

Considering that cardiovascular comorbidities represent a recognized risk factor for COVID-19 outcome, cardiovascular calcifications (calcium score) can be regarded as a surrogate radiological biomarker that can stratify the patient’s cardiovascular risk [4–6].

## Methods

The present study aims to describe gender differences in terms of clinical and radiological features and in-hospital outcomes of COVID-19 patients. The hypothesis that the different cardiovascular profile influences the different outcomes in men and women was explored through the comparison of cardiovascular calcifications.

## Study cohort

The study cohort was derived from the multicentre, retrospective, and observational sCORE COVID-19 (calcium score for COVID-19 Risk Evaluation) registry, which has been described elsewhere [6]. Briefly, the sCORE COVID-19 registry included data from 16 Italian hospitals directly involved in the COVID-19 emergency during the study period (Mar 1–Apr 20, 2020). All consecutive patients with a positive qualitative polymerase chain reaction assay for SARS-CoV-2 and a non-contrast chest CT scan performed on admission to assess pneumonia severity were included. The study population was divided into two groups according to gender (male and female), and data were analyzed for each group [7]. The local ethics of each institution committee approved the study.

## Data collection

Demographic characteristics, cardiovascular risk factors, comorbidities, and history of coronary artery disease were collected. Laboratory data included baseline admission values of hemoglobin, white blood cell count, creatinine, baseline high sensitivity troponin I (HS-TnI), lactate dehydrogenase (LDH), and C-reactive protein (CRP).

## Chest ct scan analysis

Chest CT scans were sent to the central core lab (Experimental Imaging center, IRCCS, Ospedale San Raffaele, Milano). They were analyzed by three expert cardiothoracic radiologists blinded to patients’ clinical data. Clinical and radiological data were integrated and analyzed by the coordinating center (Maria Cecilia Hospital, GVM Care & Research, Cotignola).

CT scans had been acquired with a standard non-gated chest CT protocol, using multidetector scanners with at least 16 detector rows [6]. For lung parenchyma evaluation, CTs were reconstructed at each site with a sharp kernel and visualized at the core lab using a standard lung window (width 1400 HU; center −450 HU). Analysed lung parameters included (1) semi-quantitative pneumonia scoring [no pneumonia (0%); minimal pneumonia (1–25%); mild pneumonia (26–50%); moderate pneumonia (51–75%); and severe pneumonia (76–100%)] [8]. For calcium quantification, CTs were reconstructed at each site with a soft kernel, transferred to the core lab, reformatted at a standard slice thickness of 2.5 mm without overlap or gap, and visualized using a standard mediastinal window (width 350 HU; center 40 HU). Coronary artery calcifications were visually assessed (presence/absence and number of involved vessels) and quantitatively computed. Quantification of coronary artery (CAC) was performed both with Agatston calcium scoring (CS) [9] and calcium volume (CV) methodology [10], semi-automatically, on commercial software (IntelliSpace v 8.0, Philips, The Netherlands), as follows: vascular calcifications were automatically detected as a group of adjacent pixels with an area ≥ 1 mm^2^ and a density above 130 HU. An experienced cardiothoracic radiologist labeled every calcification as belonging to coronary arteries (left main, left anterior descending, left circumflex, or right coronary artery). The interaction between cardiovascular calcifications and sex was investigated: the population was subdivided according to the coronary calcium score: absent of calcium score (CAC= 0), mild coronary calcifications (CAC 0–100), and moderate to severe coronary calcifications (CAC> 100).

## Statistical analysis

The qualitative variables are expressed as a percentage, and the differences were tested with the chi-square test. Quantitative variables are expressed as mean and standard deviation. The Student t test was used to test the statistical difference of continuous variables, adjusted according to the distribution’s possible normality.

The population was distinguished in the analysis based on biological sex (men vs. women).

The overall cohort was split based on gender and each gender further sub-categorized into survivors or non-survivors.

Continuous variables were compared with the Student t test adapted according to their distribution. Dedicated plots were constructed to compare the population’s risk profile based on gender and outcome using the relative risk ratio. Cox regression was used using “time to death after admission” as a time variable. Statistical significance of age, sex, creatinine, white blood cells, LDH, and interstitial lung involvement percentage was calculated on the univariate Cox analysis.

The choice of the included variables was empirical, considering the variable’s different domains and the preliminary data reported in the literature.

In addition to age (the most important demographic variable) and creatinine, white blood cell counts (expression of inflammation) and LDH (a marker of organ damage) were included in the model. Radiological lung injury was accounted for using the percentage of interstitial involvement categorized as greater/less than 50% of the lung parenchyma.

The univariate significance of these variables was included in a multivariate Cox regression model. The interaction of these variables with the calcium score and sex was explored by repeating the Cox multivariate in patients with mild coronary calcifications (0<CAC <100) and in those with moderate-severe coronary calcifications (CAC>100 mm^3^).

## Results

The study population included 1683 hospitalized patients with confirmed COVID-19 infection and admission chest CT. Table 1 shows the demographic characteristics and comorbidities of the overall population and two cohorts. Group A included 1131 men (67.2%) and group B 552 women (32.8%). Globally, the mean age was 67±14 years, with no statistically significant difference between the groups. In total, 910 patients (55%) had arterial hypertension, 319 (19.3%) diabetes mellitus, and 170 (11.4%) coronary artery disease with previous revascularization (percutaneous in 7.1% and surgical in 4.3%). Other prevalent comorbidities included chronic lung disease (166, 10%), chronic kidney disease, and active malignancy (86, 5.2%).

**Table 1:** Descriptive table of the population

	All	Female patients	Male patients	p-value
Demographic and clinical variables				
Number of patients, N (%)	1683	552 (32.8)	1131 (67.2)	-
Age, years±SD	67.1±14	67.8±14	67.1±13	0.3
Hypertension, n/N (%)	910 (55)	298 (55.2)	503 (54.9)	0.9
Diabetes, n/N (%)	319 (19.3)	80 (14.8)	239 (21.4)	0.001
Previous coronary stent, n/N (%)	119 (7.1)	24 (4.3)	95 (8.4)	0.002
Previous CABG, n/N (%)	51 (4.3)	7 (1.7)	44 (5.6)	0.002
Peripheral artery disease, n/N (%)	104 (6.3)	32 (5.9)	72 (6.5)	0.67
History of atrial fibrillation, n/N (%)	146 (9.2)	47 (9)	99 (9.3)	0.84
Current smoking, n/N (%)	81 (6.5)	15 (3.5)	66 (8.1)	0.002
Chronic lung disease, n/N (%)	166 (10)	65 (12.1)	101 (9.1)	0.057
History of oncological malignancy, n/N (%)	86 (5.2)	30 (5.6)	56 (5)	0.64
Baseline laboratory results				
Hemoglobin, g/dl±SD	13.6±4	13±5.9	13.8±1.9	0.002
White blood cells, n/mm^3^±SD	9988±4450	9125±4211	10392±4503	0.001
Creatinine, mg/dl±SD	1.18±0.7	0.97±0.53	1.28±0.78	0.001
CRP, mg/dl±SD	15.8±19	13.4±18	16.9±20	0.001
LDH, U/l±SD	400±250	354±196	421±269	0.001
Baseline troponin, ng/ml±SD	120±652	46±106	166±819	0.13
Oxygen saturation on air, %±SD	90±8	92±7	90±8.5	0.001
Radiological findings				
Coronary calcification (CAC), n/N (%)	994 (59.1)	281 (50.9)	713 (63)	0.001
Left main, mm3±SD	22±73	17±83	27±95	0.057
LAD, mm^3^±SD	129±245	74±183	173±328	0.001
LCX, mm^3^±SD	65±225	36±194	74.6±284	0.01
RCA, mm^3^±SD	108±402	42±182	121±460	0.001
CAC volume, mm^3^±SD	319±753	171±479	391±847	0.001
Zero calcium score, n/N (%)	508 (30.2)	224 (40.6)	284 (25.1)	0.001
CAC >100, n/N (%)	580 (34.5)	132 (23.9)	448 (39.6)	0.001
Thoracic aorta calcification, n/N (%)	1269 (75.4)	423 (76.6)	846 (74.8)	0.41
Thoracic aortic calcification, mm^3^±SD	2355±4505	2295±4600	2383±4462	0.73
Aortic valve calcification, n/N (%)	706 (41.9)	239 (43.3)	467 (41.3)	0.14
Aortic valve calcium, mm^3^±SD	174±620	129±514	194±663	0.064
Pleuric effusion, n/N (%)	252 (15)	81 (14.7)	171 (15.1)	0.81
Pericardial effusion, n/N (%)	94 (5.6)	34 (6.2)	60 (5.3)	0.47
Interstitial lung involvement <25, n/N (%)	515 (30.6)	211 (38.2)	304 (26.9)	0.001
Interstitial lung involvement 25–50, n/N (%)	694 (41.2)	190 (34.4)	504 (44.6)	0.001
Interstitial lung involvement 50–75, n/N (%)	324 (19.3)	89 (16.1)	235 (20.8)	0.023
Interstitial lung involvement >75, n/N (%)	66 (3.9)	22 (4)	44 (3.9)	0.92
In-hospital outcomes				
MACEs, n/N (%)	105 (6.2)	28 (5.1)	77 (6.8)	0.16
Stroke, n/N (%)	26 (1.5)	9 (1.6)	17 (1.5)	0.84
Peripheral arterial embolization, n/N (%)	14 (0.8)	4 (0.7)	10 (0.9)	0.73
Pulmonary embolism, n/N (%)	54 (3.2)	15 (2.7)	39 (3.4)	0.42
Acute coronary syndrome, n/N (%)	14 (0.8)	4 (0.7)	10 (0.9)	0.73
NIV without intubation, n/N (%)	291 (17.3)	74 (13.4)	217 (19.2)	0.003
Intubation, n/N (%)	207 (12.3)	43 (7.8)	164 (14.5)	0.001
Mortality in mechanical ventilated patients, n/N (%)	57 (3.4)	7 (1.3)	50 (4.4)	0.001
Hospital mortality in patients with MACEs, n/N (%)	30 (1.8)	7 (1.3)	23 (2)	0.26
Hospital mortality, n/N (%)	370 (229	94 (17)	276 (24.4)	0.001
Time to intubation after admission, days±SD	4.8±5	2±3	5±6	0.05
Time to death after intubation, days±SD	9±7	6±7	10±7	0.28
Time to death after admission, days±SD	14±8	8±7	15±7	0.07

## Sex differences

Men had a higher prevalence of cardiovascular comorbidities, including diabetes (21.4% vs. 14.8%, p=0.001), coronary artery disease with previous percutaneous and surgical revascularization (8.4% vs. 4.3%, *p*=0.002; 5.6 % vs. 1.7%, *p*=0.002 respectively), and greater smoking habit (8.1% vs. 3.5%, *p*=0.002). Conversely, women had a trend toward higher prevalence of chronic lung diseases (12.1% vs. 9.1%, *p*=0.057).

Regarding the admission laboratory data, men had higher levels of creatinine [1.28 (0.78) vs. 0.97 (0.53) mg/dl, *p*=0.001], white blood cells [10392 (SD ±4503) vs. 9125 (SD ±4211) n/mm^3^, *p*=0.001], CRP [16.9 (SD ±20) vs. 13.4 (SD ±18) mg/l, *p*=0.001], and LDH [421 (SD ±269) vs. 354 (SD ±196) mg/dl, *p*=0.001], but lower haemoglobin levels [13.8 (1.9) vs. 13.0 (5.9) g/dl, *p*<0.001] and oxygen peripheral saturation on air at admission (90% (SD ±8.5) vs. 92% (SD ±7)). No difference between the groups was noted at the baseline in the HS-TnI value [46 (SD ±106) vs. 166 (SD ±819) ng/l, *p*=0.13].

**Fig. 1:**
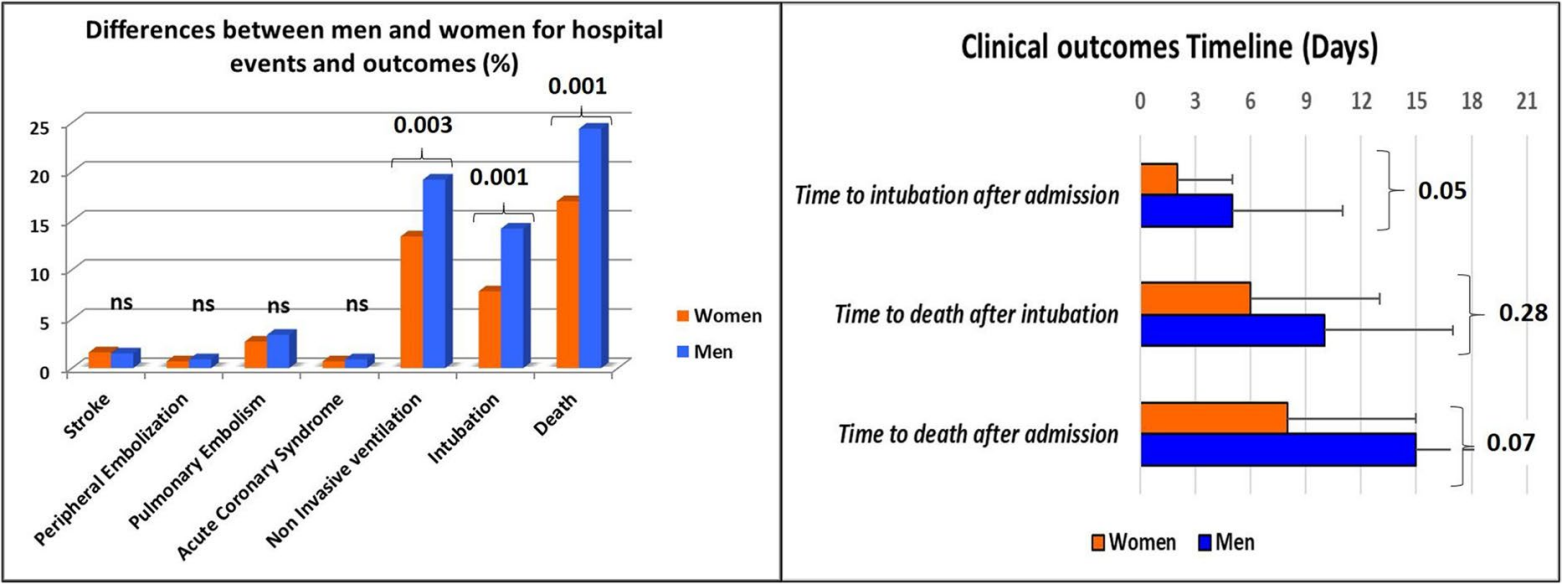
Differences between men and women for hospital events and outcomes (NIV, intubation, and death) in the graph on the left. Timeline of hospital clinical outcomes in the graph on the right. Women are represented by the orange bar, men by the blue bar

Figure 1 shows that men have a higher pneumonia extension (*p*<0.001) and required more frequently a non-invasive ventilation (19.2% vs. 13.4%, *p*=0.003) and orotracheal intubation (14.5%. vs. 7.8%, *p*=0.001). There was a significant difference in the two groups’ mortality rate, with lower values in women (24.4% vs. 17%, *p*=0.001). Since the admission, the death event tended to occur earlier in women (15±7 vs. 8±7, *p*= 0.07) (Table 2).

**Table 2:** Demographic, clinical, and radiological variables and outcomes

Variables	Survived women	Decesead women	Survived men	Decesead men	p-value (survived women vs decesead woman)	p-value (survived men vs. deceased men)	p-value (survived women vs. decesead men)	p-value (decesead women vs decesead men)
Numbers of patients, N (%)	458 (83)	94 (17)	855 (75.6)	276 (24.4)	-	-	-	-
Age, years±SD	66±14	78±9	64±12	75±10	0.001	0.001	0.001	0.031
Hypertension, n/N (%)	240 (53.7)	58 (62.4)	401 (51.9)	178 (68.5)	0.12	0.001	0.001	0.21
Diabetes, n/N (%)	62 (13.9)	18 (19.4)	160 (19)	79 (28.9)	0.17	0.001	0.001	0.23
Previous coronary stent, n/N (%)	15(3.3)	9 (9.6)	60 (7)	35 (12.7)	0.006	0.003	0.001	0.56
Previous CABG, n/N (%)	2 (3.4)	5 (1.4)	27 (4.3)	17 (10)	0.28	0.004	0.001	0.12
Peripheral artery disease, n/N (%)	24 (5.4)	8 (8.6)	38 (4.5)	34 (12.5)	0.23	0.001	0.001	0.52
History of atrial fibrillation, n/N (%)	37 (8.5)	10 (11)	54 (6.7)	45 (16.8)	0.45	0.001	0.001	0.17
Current smoking, n/N (%)	12 (3.3)	3 (4.7)	47 (7.4)	19 (10.6)	0.58	0.17	0.001	0.24
Chronic lung disease, n/N (%)	48 (10.8)	17 (18.3)	57 (6.8)	44 (16.1)	0.043	0.001	0.037	0.56
History of oncological malignancy, n/N (%)	20 (4.5)	10 (10.8)	41 (4.9)	15 (5.5)	0.016	0.68	0.53	0.045
Laboratory								
Hemoglobin, g/dl±SD	13.1±6	12.5±2	13.9±2	13.2±2	0.09	0.001	0.24	0.001
White blood cells, n/mm^3^±SD	8770±4295	10638±3242	10312±4778	10680±4048	0.001	0.26	0.001	0.92
Creatinine, mg/dl±SD	0.9±0.45	1.35±0.7	1.15±0.61	1.6±1	0.001	0.001	0.001	0.004
CRP, mg/dl±SD	12.3±15	14.9±13	16±18	16.3±11	0.11	0.81	0.001	0.41
LDH, U/l±SD	335±186	446±217	382±196	544±405	0.001	0.001	0.001	0.021
Baseline troponin, ng/ml±SD	34±90	87±145	116±757	326±986	0.034	0.14	0.008	0.25
Oxygen saturation on air, %±SD	92±5	86±9	91±8	87±9	0.001	0.001	0.001	0.7
Radiology								
Coronary calcification (CAC), n/N (%)	223 (48.7)	58 (61.7)	516 (60.4)	197 (71.4)	0.022	0.001	0.001	0.068
Left main, mm^3^±SD	10±40	48±138	20±76	39±75	0.001	0.001	0.001	0.36
LAD, mm^3^±SD	62±144	128±219	131±250	238±325	0.001	0.001	0.001	0.007
LCX, mm^3^±SD	27±138	88±275	62±233	124±266	0.003	0.001	0.001	0.46
RCA, mm^3^±SD	47±252	93±263	98±344	257±729	0.14	0.001	0.001	0.07
CAC volume, mm^3^±SD	147±455	355±743	308±745	646±1063	0.001	0.001	0.001	0.046
Zero calcium score, n/N (%)	205 (44.8)	23 (24.5)	256 (29.9)	32 (11.6)	0.001	0.001	0.001	0.004
Thoracic aorta calcification, n/N (%)	339 (74)	84 (89.4)	612 (71.6)	234 (84.8)	0.001	0.001	0.001	0.33
Thoracic aortic calcification, mm^3^±SD	1825±3978	4209±6022	1687±3462	4476±6158	0.001	0.001	0.001	0.86
Aortic valve calcification, n/N (%)	182 (39.7)	57 (60.6)	305 (35.7)	162 (58.7)	0.001	0.001	0.001	0.84
Aortic valve calcium, mm^3^±SD	79±291	353±993	138±544	364±922	0.001	0.001	0.001	0.88
Pleuric effusion, n/N (%)	61 (13.3)	20 (21.3)	124 (14.5)	47 (17)	0.047	0.3	0.16	0.24
Pericardial effusion, n/N (%)	24 (5.2)	10 (10.6)	38 (4.4)	22 (8)	0.047	0.023	0.13	0.46
Interstitial lung involvement >50, n/N (%)	76 (16.6)	35 (37.2)	170 (19.9)	109 (39.5)	0.001	0.001	0.001	0.73
In-hospital outcomes								
MACEs, n/N (%)	21 (4.6)	7 (7.4)	54 (6.3)	23 (8.3)	0.24	0.24	0.038	0.58
Stroke, n/N (%)	6 (1.3)	3 (3.2)	10 (1.2)	7 (2.5)	0.19	0.1	0.22	0.87
Peripheral arterial embolization, n/N (%)	2 (0.4)	2 (2.1)	0 (0)	10 (3.6)	0.078	0.001	0.001	0.73
Pulmonary embolism, n/N (%)	13 (2.8)	2 (2.1)	34 (4)	5 (1.8)	0.69	0.087	0.38	0.81
Acute coronary syndrome, n/N (%)	2 (0.4)	2 (2.1)	0 (0)	10 (3.6)	0.078	0.001	0.001	0.73
NIV without intubation, n/N (%)	74 (16.2)	0 (0)	217 (25.4)	0 (0)	0.001	0.001	0.001	-
Intubation, n/N (%)	36 (7.9)	7 (7.4)	114 (13.3)	50 (18.1)	0.89	0.05	0.001	0.023

## Impact of cardiovascular calcification on sex-related mortality

**Fig. 2:**
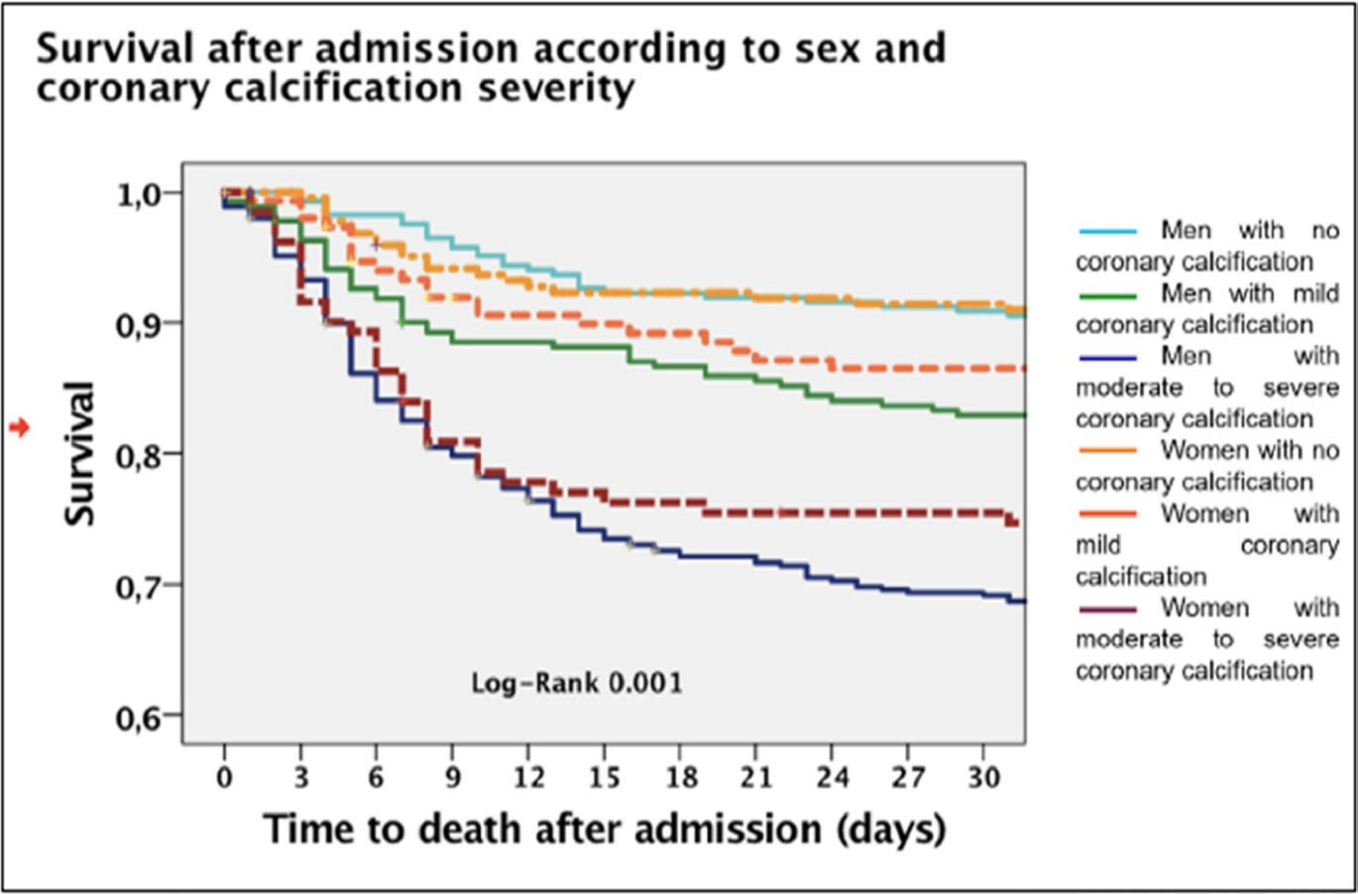
Different population mortality curves according to sex and severity of coronary calcifications (classified in the absence of coronary calcifications, mild calcifications, and severe calcifications). The dashed lines in lighter colors represent the female population. See color legend

**Fig. 3:**
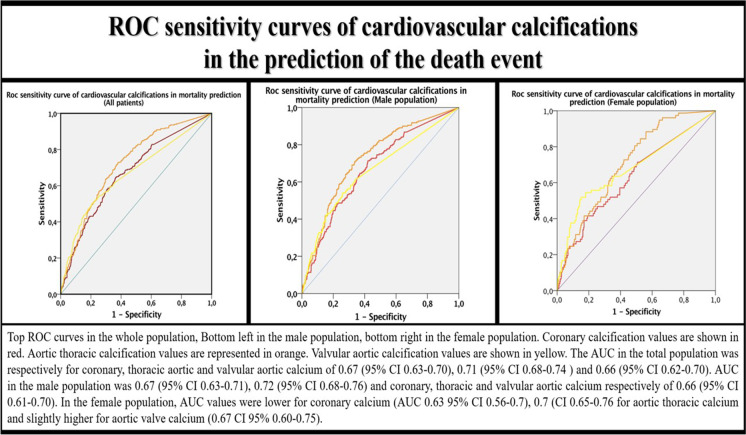
ROC curves’ analysis for cardiovascular calcifications according to sex

Men had more likely coronary calcifications (63% vs. 50.9%, *p*<0.001), with higher CAC (391±847 vs. 171±479 mm^3^, *p*<0.001) even when evaluated for every single coronary vessel (Fig. 2). No difference in terms of calcium volume of aortic valve [194±663 vs. 129±514 mm^3^, *p*=0.064] and thoracic aorta [volume (2295±4600 vs. 2383±4462 mm^3^, *p*=0.73)] was registered between two groups (Fig. 3).

**Fig. 4:**
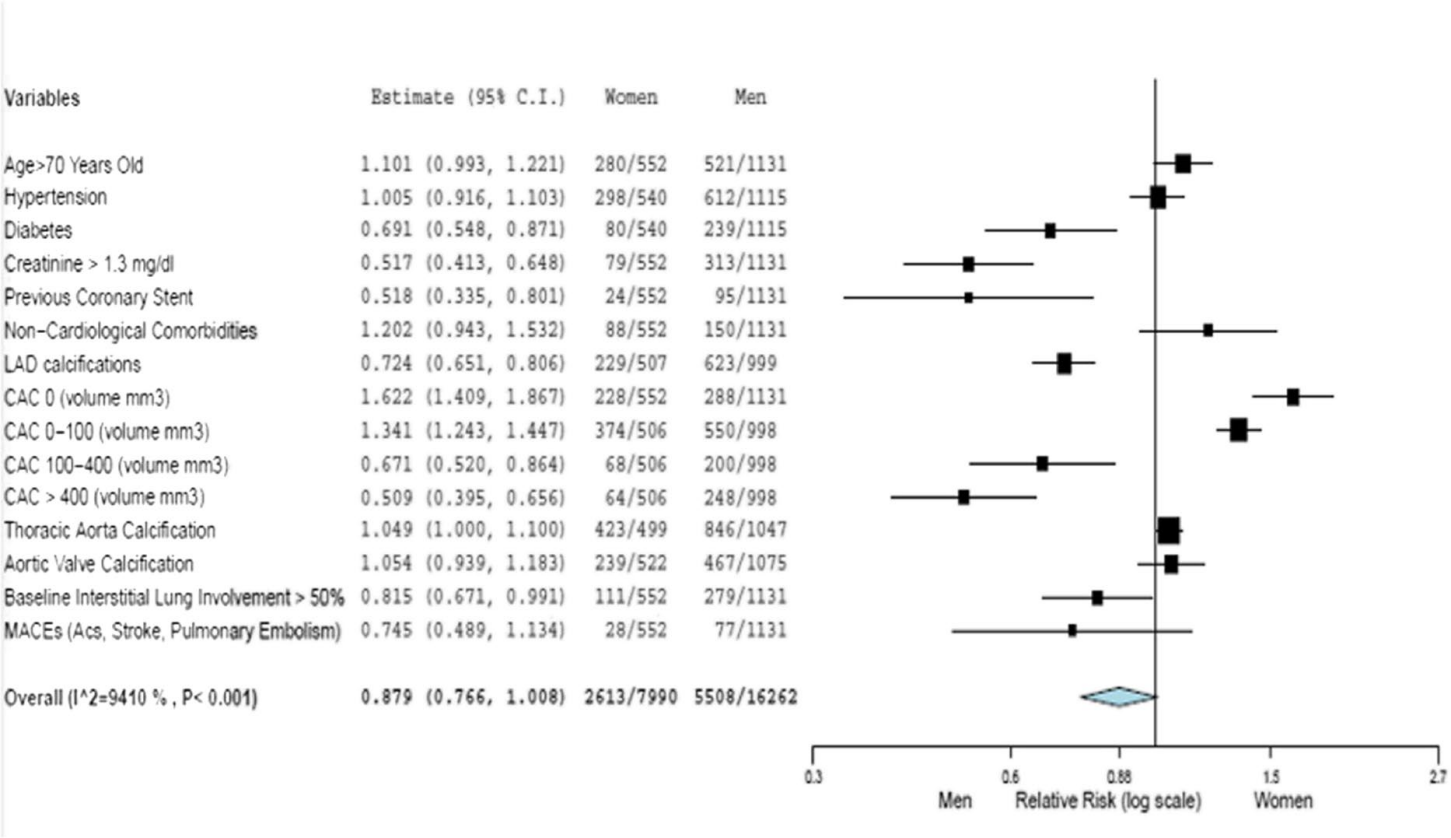
The relative risk of the clinical profile of men and women

**Fig. 5:**
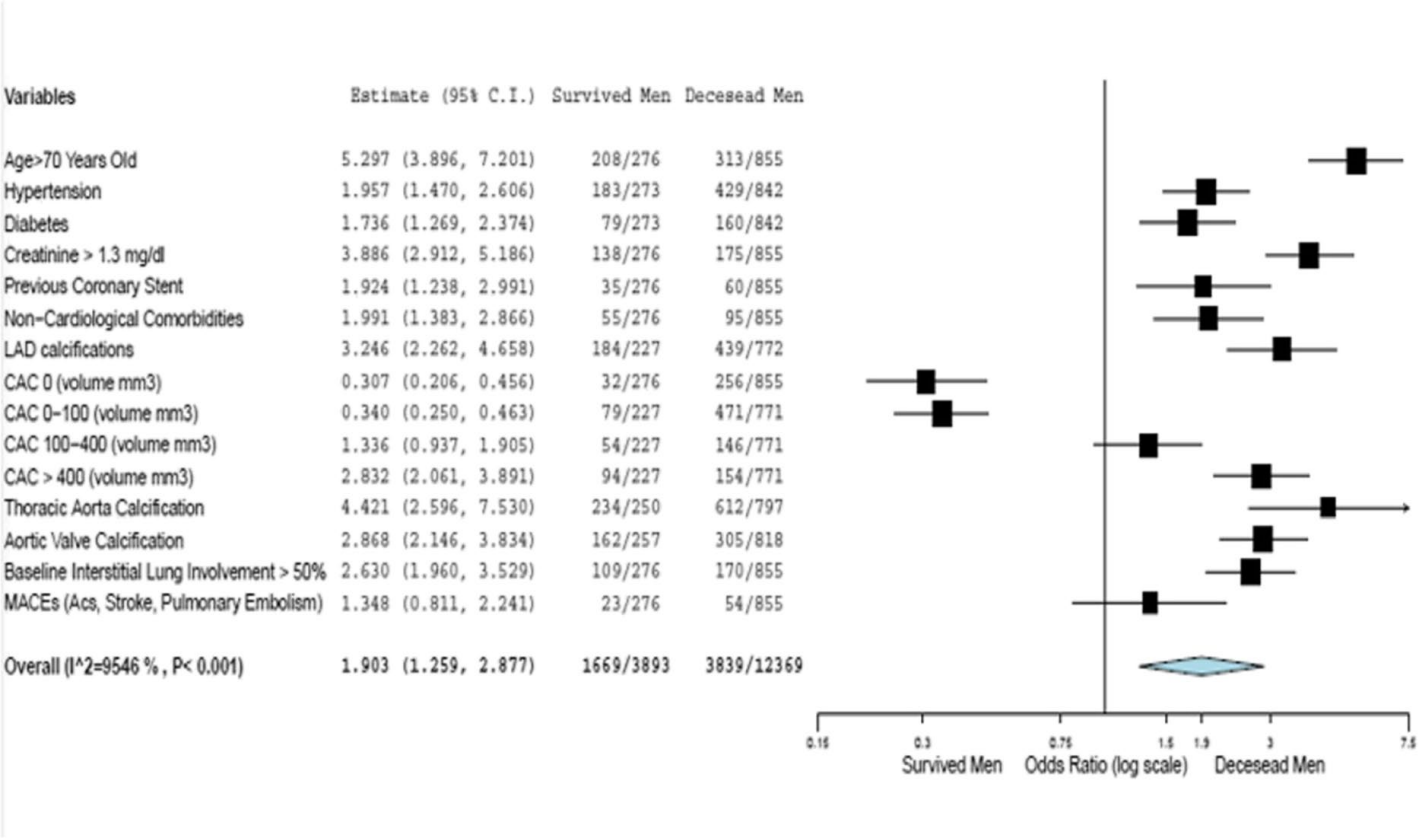
The relative risk of the clinical profile of survived men and deceased men

**Fig. 6:**
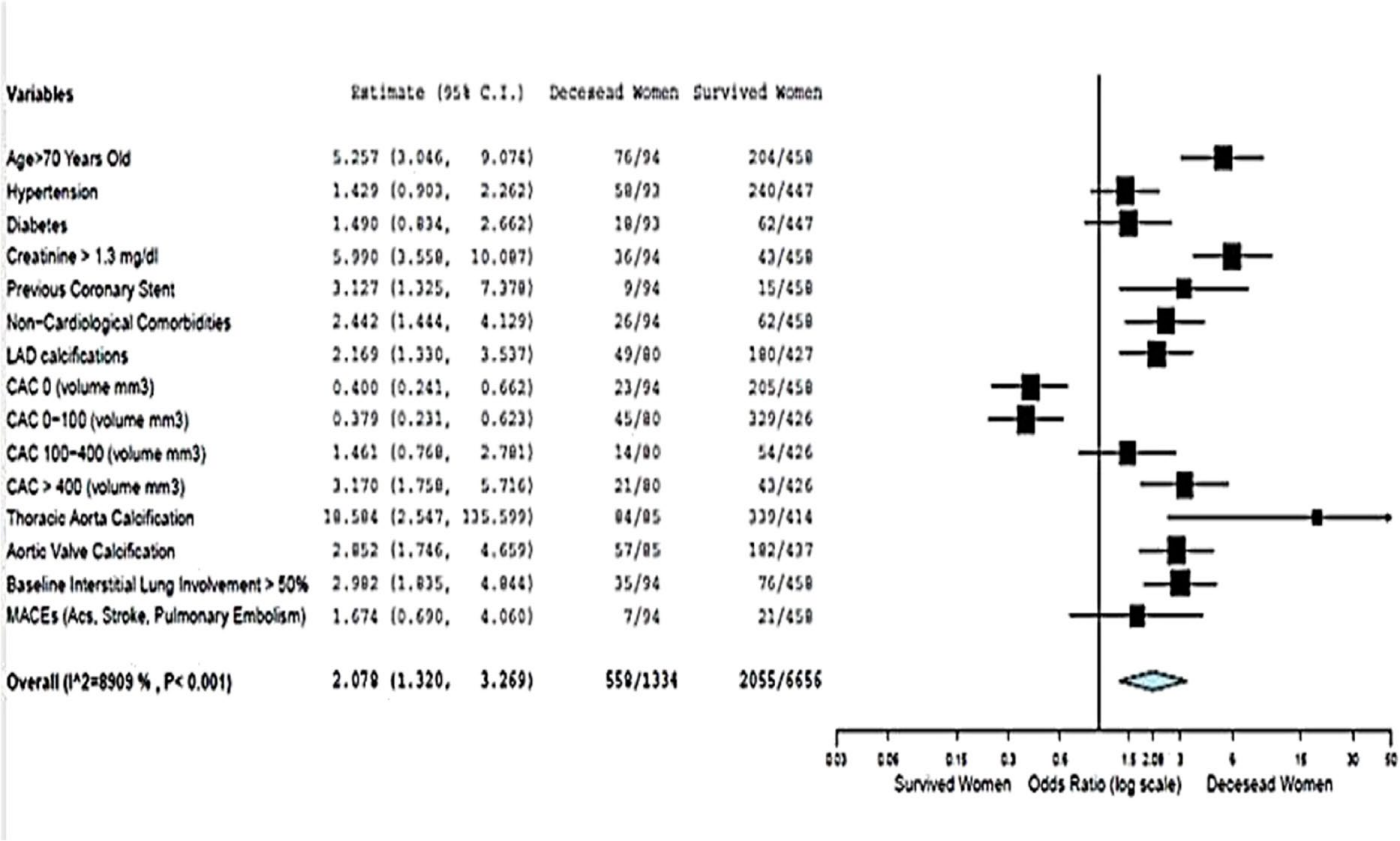
The relative risk of the clinical profile of survived women and deceased women

**Fig. 7:**
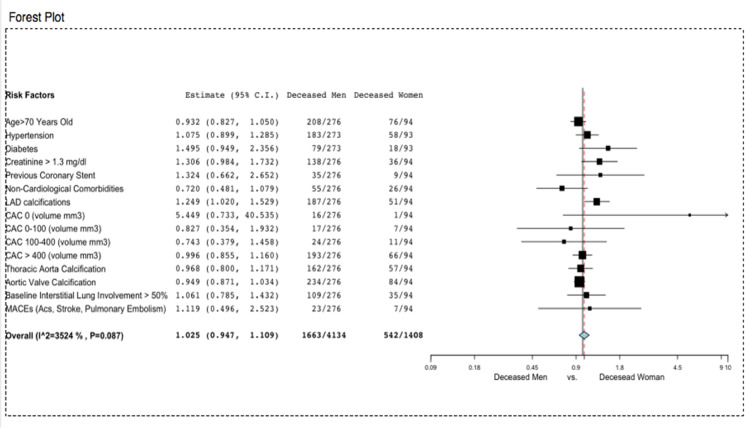
The relative risk of the clinical profile of deceased men and deceased women

The non-survived women were older than the survived ones (78±9 vs. 66±14, *p*=0.001) and had a higher prevalence of coronary artery disease (previous PCI 9.6% vs. 3.3%, *p*=0.006), chronic lung disease (18.3 vs. 10.8%, p=0.043), and chronic kidney disease. Based on chest CT measurements, non-survived women had a greater pneumonia extension (at least moderate pneumonia in 16.6% vs. 37.2%, *p*=0.001; lower well-aerated lung volume, 2234± 1290 vs. 1323 ± 864 cm^3^) (Figs. 4, 5). Similarly, the non-survived men were older than the survived ones (64±12 vs. 75±10, *p*= 0.001), with a significantly higher lung impairment and cardiovascular calcifications in terms of coronary, aortic valve, and thoracic aorta calcifications (Figs. 6, 7).

Non-survived men and women, compared to survived patients, had a higher calcium score in all the three analyzed districts (coronary, thoracic aorta, and aortic valve) (Fig. 1).

Figure 2 shows in-hospital survival curves in male and female groups subdivided according to coronary calcification severity (absent, mild, and severe). Men and women with severe calcification results have the worst outcome.

Cardiovascular calcifications had a mild-moderate sensitivity in predicting death with an AUC ranging between 0.67 and 0.72 based on the type of calcification considered. Coronary, thoracic aortic, and aortic valvular calcifications had slightly different sensitivity in predicting death between men and women.

Coronary and aortic calcifications were more sensitive in men, while aortic valvular calcifications were more significant in women (Fig. 3).

Age, female sex, creatinine, white blood cell count, LDH, interstitial lung involvement > 50%, and coronary calcium volume were significant predictors of the univariate Cox analysis.

The continuous variable with a higher HR (HR 1.52, 95% CI 1.42–1.62) was baseline creatinine. The categorical variable with a higher HR was interstitial lung involvement greater than 50%. Calcium volume was significant, with an HR of 1 (95% CI 1.0002–1.0004). The gender categorical variable was negatively associated with death with an HR of 0.66 (95% CI 0.52–0.84).

In multivariate analysis, the female sex maintained its negative predictivity for the death event (HR 0.65, 95% CI 0.50–0.92).

In patients with mild coronary calcifications, sex was significant (HR 0.68, 95% CI 0.49–0.96). In contrast, in patients with moderate-to-severe calcifications, female sex (but not age, creatinine, and LDH values) lost its statistical significance as a predictor of 30-day mortality (HR 0.89, CI 0.48–1.67).

Pulmonary radiological involvement also lost significance in patients with moderate to severe coronary calcifications (HR 1.54, CI 0.86–2.74).

Mortality curves divided by sex and severity of coronary calcifications diverged significantly (log-rank 0.001) (Table 3).

**Table 3:** Cox regression models for mortality

	Sig.	HR	95.0% CI for Exp(B)	
			Lower	Upper
Univariate Cox regression for 30-day hospital mortality				
Age (years)	0.000	1.060	1.0519	1.0677
Female sex	0.001	0.666	0.5268	0.8414
Creatinine (mg/dl)	0.000	1.524	1.4271	1.6267
White blood cells (n°/mm^3^)	0.003	1.000	1.0000	1.0000
LDH (U/l)	0.000	1.001	1.0009	1.0014
Interstitial lung involvement > 50%	0.000	2.463	1.9982	3.0359
Coronary calcium volume (mm^3^)	0.000	1.000	1.0002	1.0004
Multivariate Cox regression in all patients				
Age (years)	0.000	1.070	1.0592	1.0818
Female sex	0.014	0.685	0.5071	0.9262
Creatinine (mg/dl)	0.000	1.343	1.2158	1.4844
White blood cells (n°/mm^3^)	0.041	1.000	1.0000	1.0000
LDH (U/l)	0.000	1.001	1.0005	1.0011
Interstitial lung involvement > 50%	0.000	1.837	1.3664	2.4702
Multivariate Cox regression in patients with mild coronary calcifications (CAC volume <100 mm^3^)				
Age (years)	0.000	1.070	1.0579	1.0825
Female sex	0.028	0.688	0.4919	0.9612
Creatinine (mg/dl)	0.000	1.383	1.2404	1.5411
White blood cells (n°/mm^3^)	0.049	1.000	1.0000	1.0000
LDH (U/l)	0.000	1.001	1.0005	1.0011
Interstitial lung involvement > 50%	0.000	2.017	1.4532	2.8002
Multivariate Cox regression in patients with moderate-severe coronary calcifications (CAD > 100 mm^3^)				
Age (years)	0.000	1.078	1.0406	1.1165
Female sex	0.736	0.898	0.4816	1.6757
Creatinine (mg/dl)	0.002	1.266	1.0938	1.4665
White blood cells (n°/mm^3^)	0.065	1.000	1.0000	1.0000
LDH (U/l)	0.011	1.001	1.0002	1.0012
Interstitial lung involvement > 50%	0.139	1.543	0.8691	2.7404

## Discussion

The main findings of this sCORE-COVID-19 registry sub-analysis are that women, compared to men, (1) had lower in-hospital mortality; (2) experienced a less pronounced inflammatory reaction with a lower pneumonia extension; and (3) had a lower cardiovascular risk profile with fewer coronary calcifications.

This trend was even confirmed within the whole women group: indeed, non-survived women were older than the survived ones with a higher cardiovascular risk profile, including coronary calcifications. Our analysis is in line with the recent literature confirming that women are globally more protected from the COVID-19 adverse outcomes, having a more favorable clinical and radiological cardiovascular risk profile [8–10]. However, the protective effect of the female gender on mortality seems to be lost in women with moderate to severe coronary calcifications. Other possible reasons for sex disparities in COVID-19 outcome are the biological differences in sex chromosome genes and sex hormones that may contribute to the immune response’s different regulation. Women are functional mosaics for X-lined genes [11, 12].

The X chromosome contains a high density of immune-related genes; therefore, women generally mount more robust innate and adaptive immune responses than men.

Genes encoded on X chromosomes, and sex hormones may explain the decreased fatality of COVID-19 in women.

The angiotensin-converting enzyme 2 gene is located on X chromosomes. Men, with a single X chromosome, may lack the alternative mechanism for cellular protection after exposure to SARS-CoV-2.

As an element of novelty, calcium score, diversified into coronary calcifications, aortic valve calcifications, and thoracic aortic calcifications, plays a role in COVID-19 mortality prediction.

The coronary calcium score is a well-recognized parameter that can identify heart disease and estimate cardiovascular events’ risk. Nevertheless, calcium score provides additional information regarding total mortality risk beyond traditional risk factors [6, 10].

Calcium score should be considered a measure of arterial aging reflecting several cardiovascular risk factors’ cumulative effect.

Coronary calcium likely reveals a general biological weakness or a reduced microvascular or endothelial reserve, even more accurately than aging and other comorbidities.

Cardiovascular calcifications appear to be an intergender parameter strongly associated with a poor outcome. Indeed, even if overall mortality in COVID-19 seems lower in women, the intergender gap is not confirmed in the subgroup of patients with moderate to severe CAC [13, 14].

The analysis of cardiovascular calcifications allows to “photograph” the cardiovascular burden of every single patient at a precise moment. In contrast, cardiovascular anamnestic variables are often retrospective and may underestimate the cardiovascular burden, especially in women [7, 15].

The present study’s limitations are the retrospective analysis of the clinical and radiological data collected during the first pandemic wave peak. Therapeutical data have not been taken into account due to a standardized approach during the study period. Our study population included only COVID-19 patients who had undergone chest CT for lung assessment [16]. Therefore, these findings are potentially not reflective of all COVID-19 patients. However, it is relevant to underline that all consecutive patients by each participating center were enrolled. Moreover, our study population’s clinical, laboratory, and outcome features were consistent with those previously reported in the literature for COVID-19 [17–20].

### Limitations of the present study

In the differences in laboratory values (hemoglobin, creatinine, and white blood cells) and the values of cardiovascular calcifications, it is necessary to take into account that women have different basal values compared to men.

The comparison between survivors and deceased versus the overall population partially limits this bias. In our study, some risk factors are not available that could explain the different calcific burdens such as dyslipidemia or uric acid levels.

We have no information on chronic cardiovascular medications of these patients and therapies used in the treatment of COVID-19 disease beyond ventilation therapy.

The prophylactic use of heparin and steroids in SARS-CoV2 infection was not yet known at the time of the first wave and these data are not collected.

The outcomes of the present study concern the first region of the western world affected by the epidemic and certainly the outcomes are worse than those of other regions affected in the following weeks and compared to the other epidemic waves.

## Conclusion

COVID-19-infected women were less hospitalized than men and had more favorable outcomes. The cardiovascular burden in terms of comorbidities and coronary, thoracic aorta, and aortic valve calcifications was less represented in women [7].

However, in the multivariate analysis model, the interaction between sex and mortality was not significant in the population with moderate-to-severe coronary calcifications. Our data suggest that the protective effect of the female sex is attenuated in women at increased cardiovascular risk. In a viral infection with multiple pleiotropic cardiovascular manifestations, the protective factor of sex is lost in women with coronary calcifications (calcium score volume > 100 mm^3^).

The presumed favorable female sex bias in COVID-19 must therefore be reviewed in the context of comorbidities, especially cardiovascular ones.
